# Gliosarcoma: A Reappraisal of Neuroradiological, Surgical, Morphological, and Radiotherapeutic Characteristics of a Cohort From South Italy Single‐Center

**DOI:** 10.1155/ancp/4966984

**Published:** 2026-02-23

**Authors:** Ida Rizzuto, Antonio Ieni, Sarah Caroline Scarcella, Valentina Sciacca, Vincenzo Fiorentino, Claudio Napoli, Maurizio Martini, Antonino Germanò, Francesca Granata, Stefano Pergolizzi, Giovanni Tuccari

**Affiliations:** ^1^ Department of Human Pathology in Adult and Developmental Age “Gaetano Barresi”, University of Messina, Via Consolare Valeria 1, 98125, Messina, Sicily, Italy, unime.it; ^2^ Department of Biomedical, Dental Science and Morphological and Functional Images, University of Messina, Messina, Sicily, Italy, unime.it

**Keywords:** central nervous system, gliosarcoma, imaging, immunohistochemistry, prognosis, radiotherapy

## Abstract

Gliosarcoma (GS) is a rare and aggressive variant of glioblastoma multiforme (GBM), characterized by a biphasic histopathological pattern featuring both glial and mesenchymal components. Accounting for 2% of all GBM cases, this brain tumor is known for its poor prognosis, rapid progression, and resistance to conventional treatments. Despite advances in molecular profiling and multimodal treatment strategies, GS remains a therapeutic challenge due to their highly invasive nature and limited response to standard regimens. The purpose of this study was to characterize the neuroradiological, surgical, clinicopathological, and radiotherapeutic profiles of a cohort of patients affected by GS, including two recurrences of disease.

## 1. Introduction

Gliosarcoma (GS) is a rare and aggressive variant of glioblastoma multiforme (GBM), characterized by a biphasic histopathological pattern featuring both glial and mesenchymal components [1]. GS accounts for 1.8%–2.8% of GBMs IDH‐wildtype (IDH‐wt) [1] and typically affects adult patients with median age of 40–60 years, with a slightly higher frequency in males than females (M:F ratio 1.4–1.8:1) [1]. Most commonly, GS occurs de novo in supratentorial sites, especially in frontal, parietal and temporal areas, less frequently in posterior fossa, lateral ventricle and spinal cord; in some cases, GS can develop as a secondary form after conventional treatment of GBMs [2, 3]. Clinically, GAs often manifest with symptoms related to intracranial pressure (such as headache, nausea, vomiting, hemiparesis, drowsiness, and coma), or with focal neurological deficits depending on tumor site (such as language and personality disorders, seizures, or motor weakness) [2, 4]. Interestingly, extracranial metastases have been reported in 11% of GS compared to GMBs and other malignant glial tumors [5]; the most frequent metastatic sites are represented by lungs (72%), liver (42%), and lymph nodes (18%), followed by spleen, adrenal glands, kidney, oral mucosa, skin, and bone [6]. However, metastatic foci can be represented by both glial and mesenchymal components, but most cases show exclusively a mesenchymal/sarcomatous component [6].

Grossly, GS appears as firm, well‐demarcated masses, superficially located and weakly attached to dura [1]. While the gliomatous component is soft and presents signs of hemorrhage and necrosis, by contrast the mesenchymal component is firm and so easy to distinguish from adjacent brain tissue [1]. Histologically, a characteristic biphasic pattern, composed of featuring malignant glial and sarcomatous components within the same tumor, is usually encountered; specifically, the gliomatous part exhibits the hallmarks of GBMs, including pleomorphic astrocytes, pseudo‐palisading necrosis, mitotic activity, microvascular proliferation and, in rare cases, showing ependymal or PNET‐like differentiation [7]. On the other hand, the sarcomatous part resembles a high‐grade spindle cell sarcoma, often resembling fibrosarcoma [1], although other variety of morphologies have been described over the years (such as osteosarcoma [8, 9], chondrosarcoma [10, 11], angiosarcoma [12], rhabdomyosarcoma [13], liposarcoma [14], and pleomorphic sarcoma [15]). In addition, few reported GS cases with areas of epithelial differentiation have been rarely described [16]. By immunohistochemistry (IHC), the glial component expresses GFAP, S100, and Olig2, whereas the sarcomatous region shows positivity for vimentin, and occasionally for smooth muscle actin (SMA), desmin, or collagen type IV [1].

The prognosis of GS is poor, with a median overall survival (OS) of 9 months, compared to 15 months for IDH‐wt GBMs [5]. Currently, a combined treatment involving surgery, chemotherapy and radiotherapy has been performed [17]. The efficacy of Temozolomide (TMZ) is low in GS compared to GBMs [18]. Finally, radiation treatment (RT) after surgery demonstrated an increase in OS in GS patients (8–15 weeks longer) [19–21]. Since GSs tend to recur rapidly, a second surgical resection is planned in case of localized recurrence, although it is rarely curative; also, re‐irradiation has been considered, using hypofractionated or stereotactic techniques. On the light of all abovementioned characteristics of GS, we have thought to be of interest to perform a multidisciplinary approach to a GS series coming from a South Italy geographic area to have a current reappraisal of this rare neoplastic entity of the central nervous system.

## 2. Materials and Methods

### 2.1. Case Selection and Clinical Data

From archive files present in Department of Human Pathology of Adult and Evolutive Age, Section of Pathological Anatomy, University of Messina, A.O.U. Gaetano Martino (Messina, Italy), a single‐center retrospective cohort study was conducted on 10 cases of GS, neurosurgically obtained and histologically confirmed between 2012 and 2024; moreover, two cases of GS selected revealed tumor recurrence and they were treated as independent events. Clinical data of all patients included sex, age, Karnofsky performance status scale (KPSS) and site of disease as well as MRI and neurosurgical data were available; moreover, follow‐up (recurrence and OS) and radiotherapeutic applied protocols were also recorded.

### 2.2. Neuroradiological Features

All magnetic resonance imaging (MRI) procedures were performed in the clinical routine using a 1.5 T MRI scanner (Ingenia Philips Healthcare, Best, The Netherlands). The protocol included:

• Axial DWI single‐shot spin‐echo (SE) echo planar sequence (repetition time (TR)/echo time (TE): 3000/85 ms, Flip Angle (FA): 90°, SL: 71.6 mm, slice thickness (ST): 5 mm, field of view (FOV): 230 mm). Diffusion‐sensitizing gradients were applied sequentially in the *x*, *y*, and *z* directions with b factors of 0 and 1000 s/mm^2^. ADCs were automatically calculated by the MR scanner and displayed as corresponding ADC maps;
• Axial SE (SE) T1‐weighted sequence (TR: 633 ms, TE: 15 ms, FA: 69°, SL: 71 mm, ST: 5.50 mm);
• Axial Fast SE (FSE) T2‐weighted sequence (TR: 4848 ms, TE: 100 ms, FA: 90°, SL: 71.60 mm, ST: 5 mm);
• 3D Fluid‐Attenuated Inversion Recovery (FLAIR‐3D) sequence (TR: 5200 ms, TE: 305 ms, FA: 90°, SL: 21.44 mm);
• Postcontrast 3D T1‐weighted Gradient‐Echo (GE) sequence (TR/TE: 25/4.58 ms, FA: 30°, 1‐mm section thickness, and 230 mm FOV). A standard dose (0.1 mmol/kg body weight) of gadoteric acid (Gd‐DOTA, Dotarem; Laboratoire Guerbet, Aulnay‐sous‐Bois, France) was injected intravenously.

All images were available in digital format. Image analysis was performed on a Philips Portal Viewer. For each patient, the following imaging features were evaluated: lesion location, lesion count, growth pattern (infiltrative vs. expansive), degree of perilesional edema (mild, moderate, severe), qualitative T2‐weighted signal intensity (hypointense, isointense, hyperintense, mixed), contrast‐enhancement pattern (none, peripheral, diffuse, mixed), internal composition (solid, necrotic, mixed), extraparenchymal extension, and leptomeningeal contact. In addition, mean ADC, T2 and enhancement values were independently measured by two neuroradiologists, who drew a circular region‐of‐interest (ROI) within the tumor, avoiding regions suspected of intralesional hemorrhage or necrosis. Three sets of data were collected, including ADC, T2 signal, and enhancement evaluation. A Bland–Altman plot was used to assess agreement between the two observers and confirmed good agreement, with all intraclass correlation coefficients (ICC) exceeding 0.90.

### 2.3. Surgical Treatment

At surgery, lesions presented the macroscopic appearance resembling that of GBM, with areas of neovascularization with vascular proliferation, infiltration of the surrounding brain tissue, swelling for brain edema, and necrosis. In particular, these cases with prevalent mesenchymal component presented with firm‐hard consistence, like sarcomas, allowing for, in some cases, *en-bloc* resection of the lesions. Lesions were microsurgically resected on gross total resection (GTR) or subtotal resection (STR), according to the site of presentation and functional boundaries, with the use of intraoperative fluorescence (fluorescein, indocyanine green [ICG], and 5‐aminolevulinic acid [5‐ALA]), navigation, intraoperative cortical and subcortical stimulation, under general anesthesia or awake surgery, when applicable (Figure 1).

Figure 1Intraoperative pictures: Surgery has been performed with microsurgical technique augmented by intraoperative fluorescences (indocyanine green [ICG]) (A–C), and by the use of ultrasonic aspiration (A), navigation and direct subcortical stimulation (B). Those lesions with prevalent mesenchymal component presented with firm‐hard consistence, similar to sarcomas, and were removed also in a pieces with the use of scissure (C). At the end of the procedure GTR has been obtained with respect of functional boundaries and adjacent healthy brain tissue (D).

**Figure:**
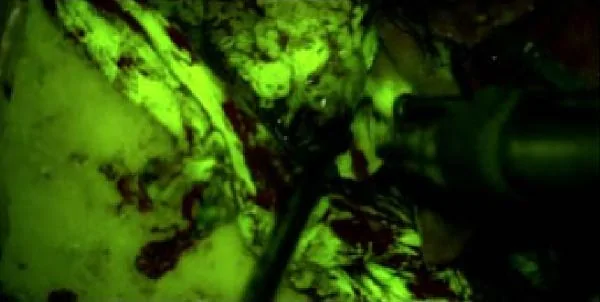
(A)

**Figure:**
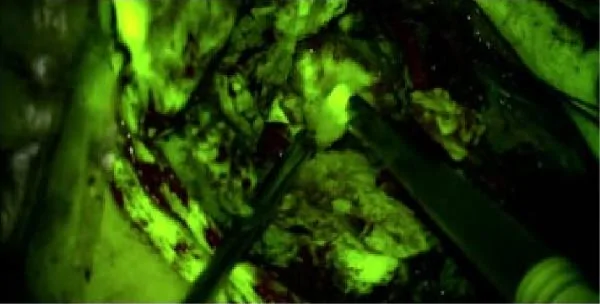
(B)

**Figure:**
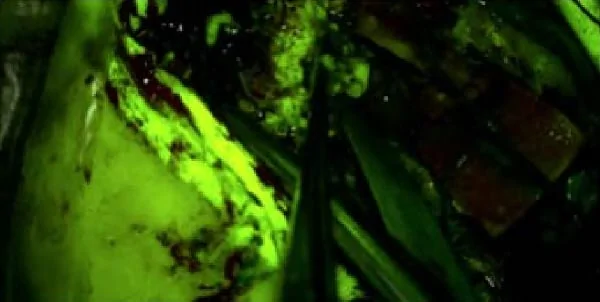
(C)

**Figure:**
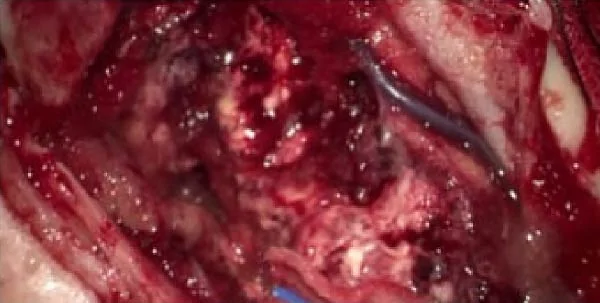
(D)

### 2.4. Histopathological and Immunohistochemical Analysis

Neurosurgically obtained neoplastic tissue specimens were fixed using 10% buffered formalin, with an exposure duration ranging from 12 to 48 h at room temperature. Subsequently, paraffin embedded tissue blocks have been obtained from which 5 µm‐thick sections were cut and routinely stained using Hematoxylin and Eosin (H&E). Parallel sections from the same tissue blocks were used for IHC. Before loading onto the Ventana BenchMark ULTRA platform, slides were deparaffinized off‐board in xylene and rehydrated through graded alcohols. Sections were then treated with 3% hydrogen peroxide for 10 min to block endogenous peroxidase activity, rinsed three times in deionized water, and incubated with normal sheep serum for 30 min at 37°C. Heat‐induced epitope retrieval was subsequently performed on the Ventana BenchMark ULTRA using Cell Conditioning 1 (CC1; alkaline Tris‐based buffer, pH 8.4), followed by on‐board incubation with the following commercially obtained primary antibodies: rabbit antibodies GFAP (clone EP672Y; ready‐to‐use), Olig2 (clone EP112; ready‐to‐use), IDH1 R132H (clone MRQ‐67; ready‐to‐use), ATRX (clone CM; ready‐to‐use), and PD‐L1 (clone SP263; ready‐to‐use); and mouse antibodies Ki‐67 (clone MIB‐1; 1:200), p53 (clone DO‐7; ready‐to‐use), vimentin (clone V9; ready‐to‐use), and EGFR (clone 3C6; ready‐to‐use). Antigen retrieval and primary antibody incubation were performed under the following conditions: GFAP EP672Y, Olig2 EP112, IDH1 MRQ‐67, ATRX CM, and PD‐L1 SP263, ULTRA CC1 64 min at 95–100°C with primary antibody incubation for 32 min at 36°C (PD‐L1 incubation 16 min at 36°C); Ki‐67 MIB‐1, ULTRA CC1 64 min at 95°C with primary antibody incubation for 32 min at 36°C; p53 DO‐7, ULTRA CC1 48 min at 100°C with primary antibody incubation for 32 min at 36°C; vimentin V9, ULTRA CC1 24 min at 100°C with primary antibody incubation for 16 min at 36°C; EGFR 3C6, CC1 60 min with primary antibody incubation for 32 min at 37°C. Following primary antibody incubation, detection was performed off‐board using a standard HRP/DAB chromogenic system: sections were washed in PBS (pH 7.2–7.4) and incubated with species‐specific HRP‐conjugated secondary antibodies (1:10,000; Abcam; ab205718 anti‐rabbit and ab205719 anti‐mouse) for 30 min at room temperature, developed with DAB, and counterstained with hematoxylin. In order to distinguish between low and high Ki‐67 as well as p53 immunoscore, we have utilized a cut‐off point of 30% and 15%, respectively. Immunohistochemical negative controls were obtained by omitting the specific antisera and substituting PBS for the primary antibody.

Two pathologists independently examined H&E and immunohistochemical sections for each sample using a Zeiss Axioskop microscope (Carl Zeiss Microscopy GmbH) at 4x–40x objective magnification, obtaining a good agreement.

### 2.5. Adjuvant Treatment

As in GBMs, the most commonly used chemotherapeutic agent was TMZ, although its efficacy in GS remains debatable. Following neurosurgical treatment, all patients underwent radiotherapy (RT), either with a conventional standard dose (60 Gy) or, in some cases, hypofractionated RT (40 Gy).

### 2.6. Statistical Analysis

The association between clinicopathological (age, sex, and site of disease), neuroradiological features, morphological and immunohistochemical profiles of GS, as well as RT treatment was subjected to univariate analysis with Fisher’s exact test. All statistical evaluations were performed using MedCalc version 10.2.0.0 (MedCalc Software). Results were considered statistically significant when *p* < 0.05.

## 3. Results

We have selected 10 patients with age ranged 44–79 years (mean age 62.87 years); one patient was female and nine were males. Two patients of this cohort were diagnosed as GS at the age of 49 and 79 years, with recurrence of disease, respectively, at the age of 50 years and 80 years. The mean KPSS at presentation was 69.5 ± 10.5. The frontal (3/10), temporal (7/10), parietal (5/10), occipital (5/10) and insular (2/10) lobes were the documented localization, even if adjacent areas were frequently involved; two cases, occurring in male patients, exhibited recurrences 1 year after surgery. Four of 10 tumors (40%) were localized in the right cerebral hemisphere, while the other six (60%) were encountered in the left one (Table 1). All cases exhibited a variable degree (from 1+ to 3+) of parenchymal perilesional oedema, associated either with expansive pattern (6/10), with infiltrative one (4/10); extra‐parenchymal extension was encountered only in three cases, while with a leptomeningeal contact of GS was found in 8/10 cases.

**Table 1:** Clinical and pathological characteristics of patients diagnosed with GS.

Characteristics	Patients (*n* = 10 including recurrences)
Mean age, years (range)	62.87 (44–79)

Sex *n* (%)	
Male	9/10 (90%)
Female	1/10 (10%)

Site of disease	
Right cerebral hemisphere	4/10 (40%)
Left cerebral hemisphere	6/10 (60%)

Number of lesions *n* (%)	
1	8 (80%)
≥2	2 (20%)

Site of lesion^a^	
Frontal	(3/10)
Temporal	(7/10)
Parietal	(5/10)
Occipital	(5/10)
Insular	(2/10)

Growth pattern	
Expansive	6/10
Infiltrative	4/10

Leptomeningeal relationship	8/10

Extra‐parenchymal extension	3/10

^a^Adjacent areas were frequently co‐involved.

### 3.1. Neuroradiological Findings

**Figure 2:**
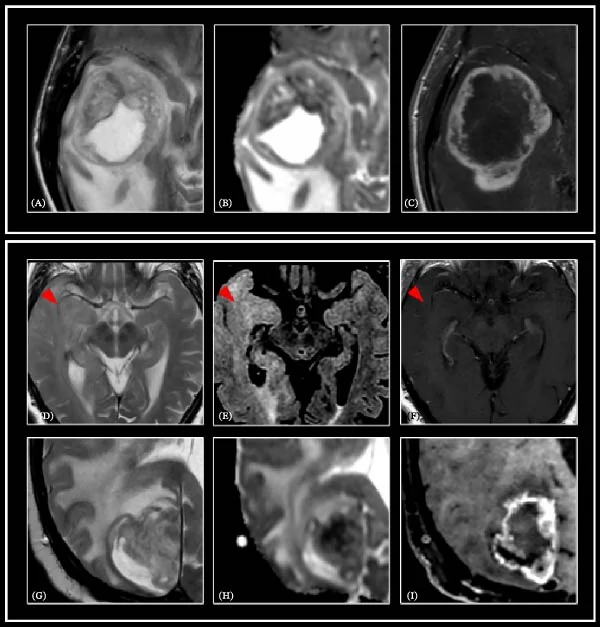
Case 1: oval‐shaped lesion in the right fronto‐temporal region, surrounded by extensive perilesional edema. The lesion shows a central necrotic component and a peripheral solid component. After Gadolinium administration, an irregular and peripheral enhancement of the lesion is observed. (A) Axial fast spin echo T2‐weighted images; (B) apparent diffusion coefficient (ADC) maps; (C) axial spin echo post‐contrast T1‐weighted images. Case 2: Baseline MRI (D, E) infiltrative lesion (red arrowhead), hyperintense on T2‐weighted images, involving both the cortex and white matter in the right temporo‐occipital and parietal region, without restriction on the ADC map. No pathological contrast enhancement is observed after Gadolinium administration (F). Recurrent case at the follow‐up MRI: note the global increase in the extent of the infiltrative lesion, with signs of tissue swelling. Evidence of a newly appearing solid component in the right temporo‐occipital region, with heterogeneous signal on T2‐weighted sequences (G), apparent diffusion coefficient (ADC) maps (H); after gadolinium administration, an intense enhancement of the solid part of the tumor is detected (I).

ADC was sampled in all cases, yielding a mean value of 0.97 × 10^−3^ mm^2^/s (range 0.62–1.26), while in two cases of GS recurrence the men value was 0.71 × 10^−3^ mm^2^/s. In detail, all GS primary tumors showed a higher mean ADC (0.97 × 10^−3^ mm^2^/s) than recurrences (0.71 × 10^−3^ mm^2^/s), specifically, the comparison between ADC value in primary and recurrent GS was 1.15 × 10^−3^ mm^2^/s versus 0.71 × 10^−3^ mm^2^/s. T2 measurements revealed a hyperintense/mixed pattern in 6 patients, onea hyper‐/isointense appearance and three characterized by mixed pattern. The overall mean T2 value for primary GS was 329.42 (range 94.41–898.42), while the corresponding mean T2 value for GS recurrence was 277.53. Enhancement patterns were mixed in six cases (60%) and peripheral in four (40%) (Figure 2). The mean enhancement value was 606.14 (primaries ≈605.11; recurrences ≈610.28) (Figure 2). Tissue composition analyzed by neuroradiology showed a mixed appearance in 6 tumors (60%), necrotic in three (30%) and solid in one (10%). Finally, 8/10 GS cases exhibited the leptomeningeal contact, while only three cases revealed an extra‐parenchymal extension. All MRI characteristics are summarized in Table 2.

**Table 2:** Neuro‐radiological characteristics of GS patients.

Characteristics	Value
ADC (solid component)	7/10

ADC mean value primary GS	0.97 × 10^−3^ mm^2^/s

ADC mean value recurrent GS	0.71 × 10^−3^ mm^2^/s

T2	
Hyperintense/mixed pattern Hyper‐/isointense appearance Mixed	6/10
	1/10
	3/10

T2 mean value	
Primary GS	329.42
Recurrent GS	277.53

Pattern of enhancement	
Mixed	6/10
Peripheral	4/10

Enhancement mean value	
Primary GS	605.11
Recurrent GS	610.28

### 3.2. Histology and Immunohistochemical Profile

**Figure 3:**
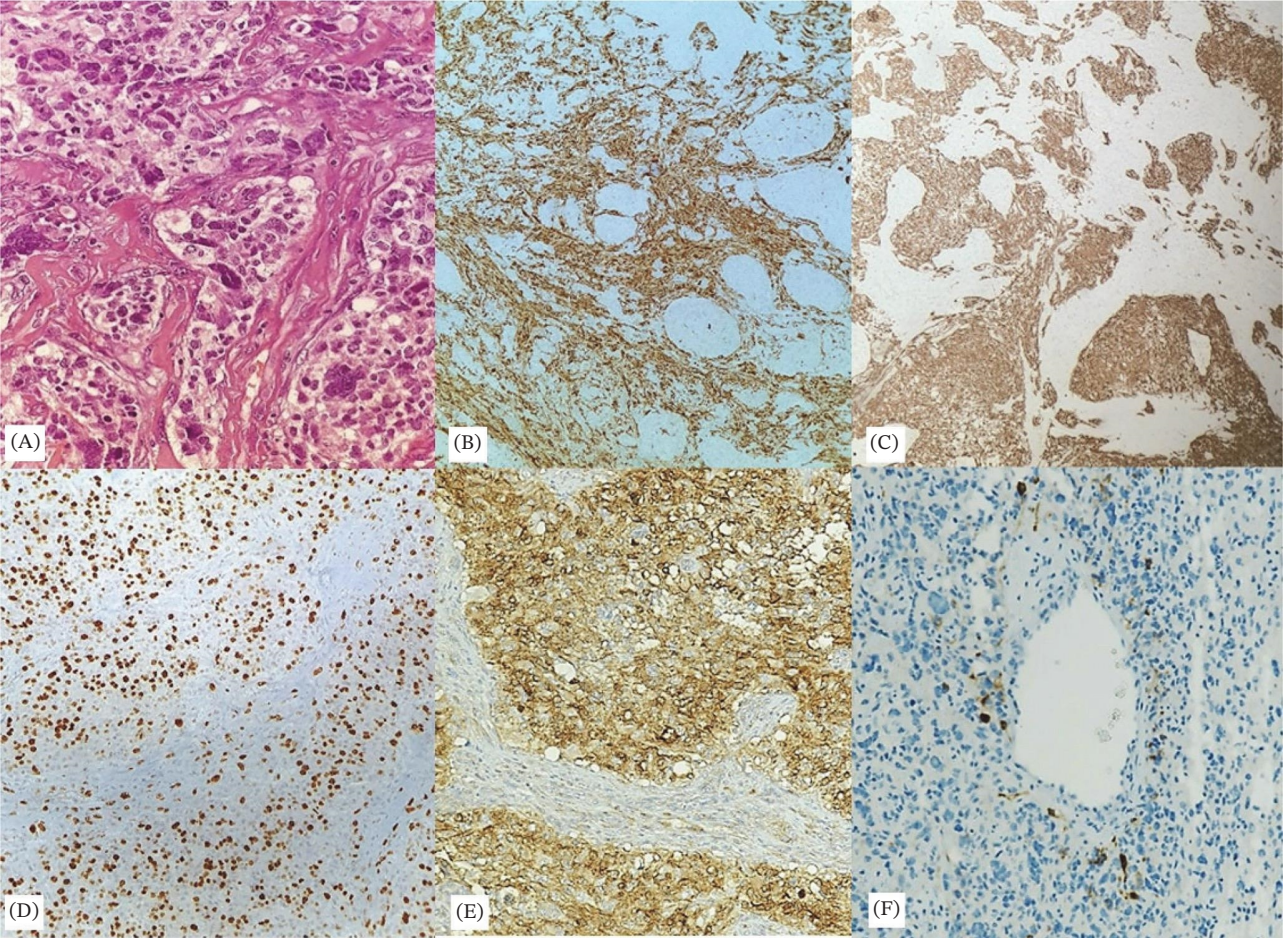
Biphasic pattern of GS showing admixture of isles made up of bizarre glial cells with extreme pleomorphism intermingled with spindle sarcomatous elements (A, 40×). Sarcomatous cells marked by vimentin (B, 10×). Glial islands showing GFAP immunoreactivity (C, 10×). Ki‐67 proliferating index (D, 20x). Diffuse and intense EGFR immunoreactivity in glial cells (E, 40×). Spotty expression of PD‐L1 in spindled elements in perivascular arrangement (F, 20×).

H&E routinely stained sections showed typical morphological features of GS, revealing an admixture of glial and mesenchymal components (Figure 3A). Immunohistochemical analysis revealed sarcomatous elements expressed vimentin (Figure 3B), while GFAP (Figure 3C), Olig2 and ATRX were constantly positive in the glial component, while these markers were constantly negative in mesenchymal cells. The growth fraction, determined by counting Ki‐67 reactive cells, was elevated, with a mean value in primitive GS 31.25%, while in recurrent cases was 47.5% (Figure 3D). All cases, including recurrences, showed a constant EGFR expression (Figure 3E), the latter being largely expressed in glial cells. Only two cases, characterized by recurrence, showed focal/spotty PD‐L1 expression in mesenchymal elements (Figure 3F), while other GS cases were PD‐L1 negative. Finally, a mean value of 21.87% in p53 expression was encountered in primitive GS, with a higher rate (55%) in recurrent GS cases.

### 3.3. Radiotherapeutic Treatment

**Figure 4:**
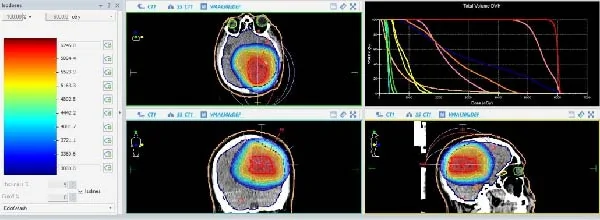
Treatment plan for a patient with GS represented in his three projections (axial, sagittal, and coronal) showing the dose’s distribution and his related dose‐volume histogram (DVH).

For every patient affected by GS, TC and MR scans were utilized to contour gross tumor volume (GTV) and a treatment plan was created using a treatment planning system (TPS) that generated a dose‐volume histogram (DVH) for better dose delivery coverage (Figure 4). All patients were treated in a LINAC using volumetric modulated arc therapy technique (VMAT). Although, the radiotherapeutical approach is generally based on the patient’s age (< or >65 years), we have chosen an hypofractionated or classic radiotherapy mainly on KPSS. Consequently, we have treated three patients, one of which was GS recurrent, with hypofractionated radiotherapy (Group 1) and seven, one of which had recurrence, with classic radiotherapy (Group 2); then, the OS was 10 months in Group 1, while in Group 2 was 4 months.

In addition, according to Stupp’s protocol, both groups were treated with TMZ in six cycles.

### 3.4. Statistical Data

Univariate analysis showed statistically significant associations with OS for Ki‐67 and p53, while enhancement value and RT approach showed a trend toward association with OS (*p* slightly above 0.05) (Table 3).

**Table 3:** Univariate analysis of variables associated with overall survival (OS).

Parameter	Value	No.	OS (months)	*p*	HR	95% CI
Enhancement	≤606.14 >606.14	6/10 4/10	7.33 6.75	0.0570	6.29	0.9–41.9

Ki‐67	≤30% >30%	3/10 7/10	3 8.86	0.0385	7.75	1.11–53.82

p53	≤15% >15%	2/10 8/10	2 8.38	0.0065	18.82	2.27–155.98

Radiotherapy	Hypofractionated Classic	3/10 7/10	10 4	0.0588	4.85	0.94–24.99

## 4. Discussion

In this single‐center South Italian cohort of GS, we integrated neuroradiological, surgical, histopathological/immunohistochemical, and RT data to provide a multidisciplinary reappraisal of this rare entity. Across 10 GS cases (including two surgically confirmed recurrences analyzed as separate events), lesions most frequently involved supratentorial regions and commonly showed marked perilesional edema and leptomeningeal contact. Quantitatively, recurrent GS showed lower mean ADC values compared with primary tumors, suggesting increased cellularity at relapse, and exhibited higher proliferative activity (Ki‐67) and higher p53 expression. Histologically, all cases displayed the characteristic biphasic pattern, with a glial component immunoreactive for GFAP/Olig2/ATRX and a sarcomatous component highlighted by vimentin. EGFR immunoreactivity was consistently observed, whereas PD‐L1 expression was only focal/spotty in a minority of cases. In univariate analysis, Ki‐67 and p53 were significantly associated with OS, whereas enhancement value and RT approach showed a trend, supporting the potential clinical utility of integrating imaging, pathology, and treatment parameters in GS risk stratification.

GS is a diagnostic challenge being uncommon, heterogeneous and subtly overlapping with GBM in imaging features, frequently displaying pronounced radiological heterogeneity and consistent signs of clinical aggressiveness. In our cohort, about 60% of the primary GS exhibited an expansive pattern, while 40% revealed the infiltrative one, including two recurrent cases; a pronounced perilesional edema is always observed.

Regarding imaging, GS shares several common radiological features with other high‐grade gliomas, although some of their characteristics are peculiar. Typically, resembling GBMs, GS presents on MRI as peripheral intra‐axial masses with predominant supratentorial location, heterogeneous enhancement, central necrosis, tendency to contact dural, pial or ependymal surface, and frequent extraparenchimal spread [22]. On T1‐weighted imaging, the tumor appears hypo‐ to isointense, while T2/FLAIR sequences show mixed signal intensity due to the biphasic histology (glial and sarcomatous components). Post‐contrast T1‐weighted images reveal strong, irregular peripheral enhancement with possible dural tail sign, contributing to diagnostic trouble with other high‐grade gliomas or brain metastases. Peritumoral oedema is common, but it may be less pronounced than in conventional GBMs, whereas the presence of an eccentric tumor cyst with a salt‐and‐pepper sign, well‐demarcated margins and hemorrhage are more frequently found in GS rather than in high‐grade gliomas [23].

It is well known that ADC is commonly used as a DWI parameter for estimation of tumor cellularity. The dual glial and sarcomatous histology of GS and its great amount of necrosis may explain the higher mean ADC values compared to other solid, highly cellular lesions. Moreover, recurrences exhibited lower mean ADC (0.71 × 10^−3^ mm^2^/s) than primaries (0.97 × 10^−3^ mm^2^/s), suggesting greater cellularity and reduced extracellular matrix, hallmarks of a more aggressive phenotype. In our study, the overall ADC range (0.62–1.26 × 10^−3^ mm^2^/s) aligns with published data, but the primary–recurrence shift highlights the potential prognostic value of serial ADC monitoring. Furthermore, the mixed enhancement remained the predominant pattern in both primaries and recurrences, yet recurrences showed slightly higher mean enhancement intensity (≈605.11 vs. ≈610.28), possibly reflecting vascular redistribution or increased stromal fibrosis. In univariate analysis, enhancement value showed a trend toward association with OS (*p* = 0.057). In addition, the reported leptomeningeal contact in 80% of cases and the extra‐parenchymal extension in 30% confirms the GS predilection for superficial spaces.

Our morphological analysis of GS cases clearly documents the double tissue components, constituted by intermingled glial and sarcomatous elements. Subsequently, a peculiar immunohistochemical biphasic histogenetic profile has been documented by GFAP and vimentin. With respect to proliferative and cell‐cycle markers, Ki‐67 and p53 were significantly associated with OS in univariate analysis (Ki‐67, *p* = 0.0385; p53, *p* = 0.0065). Notably, in our descriptive comparison, recurrence specimens also showed higher Ki‐67 and p53 expression than primary tumors, consistent with a more aggressive phenotype at relapse. Consistent with our results, literature data have documented a more diffuse p53 expression in GS also in comparison to GBM [5, 24]. However, it is well known that GS has a different molecular profile from GBMs [24]; in fact, EGFR amplification is uncommon in GS (12%), while it occurs approximately in 40% of GBMs [24]. Nevertheless, PTEN, TP53, TERT, RB1, STAG2, and NF1 mutations are instead frequent in GS and rare in GBM [25]. Moreover, some alterations overlap between GS and soft tissue sarcomas (TP53, TERT, NF1, CDKN2A/N2B, and RB) [5]. Finally, in few cases GS is related to hereditary cancer syndromes such as Lynch Syndrome with mutations of MSH2 [26]. In our cohort, we have found unexpected intense, diffuse expression of EGFR, even if literature reported low rates of EGFR mutations in GS [5]. However, EGFR is known to be an oncogenic driver in 40% of IDH wild type GBMs [25] and consequently a potential candidate for target therapy in GBM, though there are still no studies on GS due to low rates of EGFR amplification found in most cohorts.

New therapeutic strategies for GS are currently ongoing. In detail, a phase II clinical trial (NCT02798496: CAPTIVE/KEYNOTE‐192) for GBM and GS patients is evaluating the combination of DNX‐2401, an oncolytic adenovirus, with the anti‐PD‐1 antibody pembrolizumab, demonstrating a median OS of 12.3 months, compared with the OS of standard treatment of 7.2 months [27]. Programed cell death ligand‐1 (PD‐L1) expression has been reported in >60% of high‐grade gliomas and it seems to play a crucial role in glioma biology, although there is still poor and contrasting literature data on PD‐L1 role and/or PD1/PD‐L1 expression in GBM and even more so on GS [28–30]. Nevertheless, in our cohort, only 2 cases showed focal PD‐L1 expression in sarcomatous cells, whereas PD‐L1 immunoreactivity was not present in mesenchymal component or in glial one, thus challenging conventional classification of GS as a high‐grade form of GBM and suggesting potential failure of immunotherapy targeting PD1/PD‐L1 axis. The limited/absent PD‐L1 expression observed in our cohort suggests that immune‐checkpoint blockade may not be uniformly effective in GS. Notably, in gliomas, autophagy‐related programs are increasingly regarded as modulators of stress tolerance and therapy resistance and have been discussed in relation to glioma stemness and the tumor immune microenvironment, providing a biological rationale for exploring combination strategies in refractory disease [31].

The STUPP protocol has been considered the standard of care for the treatment of high‐grade gliomas since its publication in 2005 and it has led to significant survival improvements. Analogically classic STUPP protocol, including surgery, radiotherapy, and TMZ, has been utilized as the landmark in GS patients [17]. In fact, this multimodal treatment has been applied, although results were discouraging with lower mean OS in GS, compared to patients diagnosed with GBM. Interestingly, we have observed that radiotherapy modality could influence prognosis, being classic associated to shorter OS compared to hypofractionated one, even if clinical status of patients prior to the choice of treatment could affect survival independently from modality of RT. Overall, the RT approach showed a trend toward an association with OS, while Ki‐67 and p53 were significantly associated with outcome, thus representing markers of neoplastic aggressiveness.

While our data provide additional insight into prognostic factors in GS, some limitations should be acknowledged. Specifically, our study’s retrospective design, small sample size, and single‐center setting may reduce generalizability and statistical power. In addition, because recurrence specimens were included as separate events, some degree of non‐independence cannot be excluded. Larger multicenter studies incorporating comprehensive molecular profiling are therefore warranted to validate our findings and refine prognostic models in GS.

Notwithstanding these limitations, our study has several strengths. Given the rarity of GS, establishing a 10‐case single‐center cohort with paired neuroradiological metrics, centralized histopathological/immunohistochemical review, and detailed RT data represents a meaningful contribution to a sparse literature, where multidisciplinary radiology–pathology studies remain limited. In addition, imaging evaluation was performed using a standardized MRI protocol with independent measurements by two neuroradiologists and high interobserver agreement. Finally, histological and immunohistochemical assessment was independently reviewed by two pathologists, supporting the internal consistency of diagnostic classification and biomarker scoring and strengthening the reliability of the integrated radiology–pathology correlations.

## Author Contributions

Ida Rizzuto, Claudio Napoli, Francesca Granata, Antonio Ieni, and Giovanni Tuccari developed the study design and drafted the manuscript. Ida Rizzuto, Claudio Napoli, Sarah Caroline Scarcella, Valentina Sciacca, Vincenzo Fiorentino, Claudio Napoli, Maurizio Martini, and Antonino Germanò were involved in data acquisition and statistical analysis. Antonio Ieni, Francesca Granata, Antonino Germanò, Stefano Pergolizzi, and Giovanni Tuccari reviewed the manuscript. Antonino Germanò, Francesca Granata, Stefano Pergolizzi, and Giovanni Tuccari confirm the authenticity of all the raw data.

## Funding

No funding was received. Open access publishing facilitated by Universita degli Studi di Messina, as part of the Wiley ‐ CRUI‐CARE agreement.

## Disclosure

All authors have read and approved the final version of the manuscript.

## Ethics Statement

The analysis was conducted according to the Good Clinical Practice guidelines and the Declaration of Helsinki (1975, revised in 2013). Before surgical procedures, all patients provided written, anonymized and informed consent. Pathology reports and medical records were thoroughly reviewed. Patient initials and other personal identifiers were removed from all images. The Institutional Review Board of the University Hospital of Messina (Messina, Italy) approved the present study (approval no. N. 47/19; May 2, 2019).

## Consent

Written informed consent was obtained from all patients for the publication of their data.

## Conflicts of Interest

The authors declare no conflicts of interest.

## Data Availability

The data generated in the present study may be requested from the corresponding author.
